# Dynamics Calculations of the Flexibility and Vibrational Spectrum of the Linear Alkane C_14_H_30_, Based on Machine-Learned Potentials

**DOI:** 10.1021/acs.jpca.4c06943

**Published:** 2024-12-03

**Authors:** Chen Qu, Paul L. Houston, Riccardo Conte, Joel M. Bowman

**Affiliations:** † Independent Researcher, Toronto, Ontario M9B0E3, Canada; ‡ Department of Chemistry and Chemical Biology, Cornell University, Ithaca, New York 14853, United States; § Dipartimento di Chimica, Università degli Studi di Milano, via Golgi 19, Milano 20133, Italy; ∥ Department of Chemistry and Cherry L. Emerson Center for Scientific Computation, Emory University, Atlanta, Georgia 30322, United States; ⊥ Department of Chemistry and Biochemistry, Georgia Institute of Technology, Atlanta, Georgia 30332, United States

## Abstract

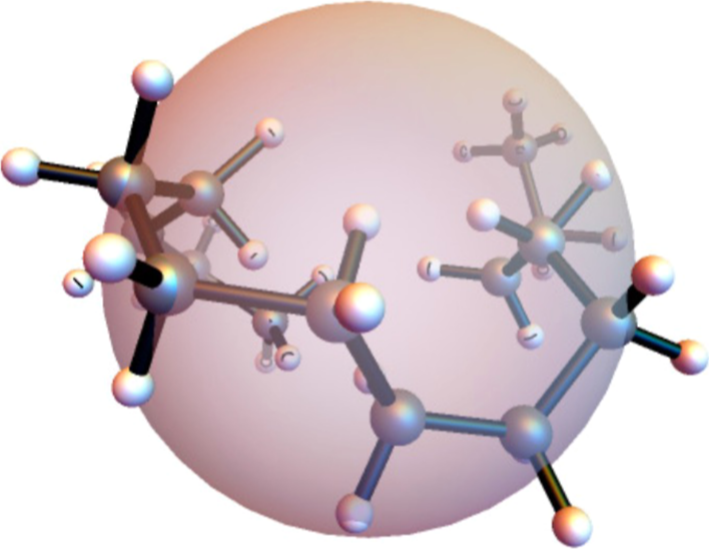

Hydrocarbons are the central feedstock of fuels, solvents, lubricants, and the starting materials for many synthetic materials, and thus the physical properties of hydrocarbons have received intense study. Among these, the molecular flexibility and the power and infrared spectroscopies are the focus of this paper. These are examined for the linear alkane C_14_H_30_ using molecular dynamics (MD) calculations and recent machine-learned potentials. All MD calculations are microcanonical and start at the global linear minimum. The radius of gyration, the number of gauche bond conformations and the distributions of all C–C distances are reported as a function of the total internal energy and as a function of time. These are compared to the power spectra and to the double harmonic spectra of stationary points. Spectral features of the double harmonic spectra smoothly track structural differences, measured by the number of gauche conformations in the molecule. Preliminary calculations using the quantum local mode model for the CH-stretch are presented and satisfactorily capture anharmonic effects.

## Introduction

Hydrocarbons are important starting materials for industrial and commercial products. Their combustion is substantially responsible for current global climate change. Consequently, there has been much study not only of their chemical properties but also of their physical and molecular properties. Among these properties, this paper focuses on their molecular flexibility, their infrared spectroscopy, and the relationships between them. Several aspects of the flexibility and spectroscopy have been reported previously.^1−7^ The temperature dependence of the experimental IR spectra of *n*-alkanes up to *n*-dodecane in spectral range 2500–3400 cm^–1^ was reported in 2007.^3^ Somewhat later, the experimental 298 K gas-phase IR spectra of a series of linear alkanes ranging from butane to tetradecane were reported at 298 K in both that same spectral range and in the range 1300–1600 cm^–1^.^4^ Raman spectra in range 100–500 cm^–1^ were also reported for *n*-alkanes ranging from 13 to 21 carbon atoms.^5,7^

We recently reported two permutationally invariant polynomial (PIP)^8−11^ potentials for the linear alkane *n*-C_14_H_30_.^12^ These were fits to roughly 253,000 B3LYP energies ranging from 0 (the energy of the global minimum) to 80,000 cm^–1^. The mean absolute error for energies is 86 and 43 cm^–1^ for these potentials. We note a recent atom-centered neural network for hydrocarbons has also been reported, with the aim to study strain and failure of knots in polyethylene.^2^ This potential was trained on a variety of hydrocarbons, using 3334 DFT energies, with a stated “average training error of 2.58 meV/atom”. For C_14_H_30_ this would correspond to an error of 915 cm^–1^, which is much larger than the error we reported.^12^ This is not surprising given that the goal of the NN potential is apply to alkanes, alkenes and alkynes, in contrast to the PIP potentials which apply only to alkanes. Another recent paper reports an interaction potential for molecules containing carbon chains with up to 7 carbon atoms.^13^

The goals of this paper are to explore the vibrational power spectra and double harmonic spectra of this alkane as the internal energy is increased and thus as the flexibility of the molecule increases. Three standard methods of flexibility characterization are used: the probability distribution of carbon–carbon distances, the radius of gyration and the number of gauche conformations. The change in each of these is described as a function of time and energy. Using the trajectories, we also calculate the power spectra as a function of energy by calculating the Fourier transform of the velocity autocorrelation function. As well, at each of 200 geometries corresponding to stationary points, we calculate the double harmonic spectrum at the DFT/B3LYP level. These power spectra and double harmonic spectra are then compared to the spectrum of harmonic eigenvalues for the global minimum structure on the PES as well as to experimental spectra.

## Methods

### Molecular Dynamics

We use our own software^14,15^ to run microcanonical (NVE) trajectories. These were calculated using a fragmentation PES (F-PES) and employing a time step of 5 au, or about 0.121 fs per step, for 10,000 steps.^12^ In all applications, trajectories were initiated at the global minimum of the potential, and the total energy was specified and distributed randomly among the kinetic energies of all atoms. The total angular momentum was adjusted to be zero for each trajectory. Properties were obtained at total energies of 5000, 15,000, 25,000, 35,000, 50,000 and 80,000 cm^–1^. At each energy, batches of trajectories were propagated with a recording of the energy, geometry, velocities, and gradients at every 10 steps. The number of trajectories at each of the above-listed energies was 100, 100, 98, 90, 240, and 154. We use the MD trajectories mainly for the calculation of the vibrational density of states via the standard calculation of power spectra. We also use the trajectories to determine the distribution of structures, with a focus on the distribution of C–C distances, the radius of gyration, and the number of gauche conformations.

### Power Spectra

To obtain the classical power spectra, we employ an open source web platform,^16^ recently developed at University of Milan. This enables the calculation of the power spectra starting from a standard molecular dynamics trajectory. The output of a standard trajectory is first uploaded to the web platform. Then, calculation of the power spectrum is performed online using the following well-known expression based on the time averaged Fourier transform of the Cartesian velocity autocorrelation function^17−19^ relative to a dynamics of duration *T*
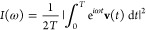
1*I* indicates the density of vibrational states, which is the observable related to a power spectrum. Power spectra report all frequencies of vibration and, differently from IR spectra, are not subject to selection rules and dipole transition strengths. This explains why power spectra can get extremely crowded when the dimensionality of the system increases. In such instances, and C_14_H_30_ is one of them, the time averaged expression is very useful. The squared modulus appearing in eq 1 allows one to get a positive-definite power spectrum and a better signal-to-noise ratio, improving readability of the spectrum and spectroscopic features of larger systems.

While this classical MD approach is very general, results depend on the energy of the evolved trajectory and some well-known issues may arise when relating the results to experiment, where quantum anharmonic effects can be significant. We discuss these briefly when we present comparisons of the present calculations of the power spectra to experimental IR and Raman spectra.

Standard double harmonic IR spectra are also calculated for a variety of stationary points on the C_14_H_30_ PES as well as for the “linear” global minimum. These are directly comparable to experimental IR spectra, with the usual caveats, which we recap below.

### Shape and Flexibility Characterization

**Figure 1:**
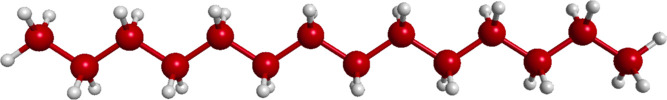
Global minimum structure of C_14_H_30_.

We have used three standard methods to characterize the shape and flexibility of C_14_H_30_: the probability distribution for C–C distances, the radius of gyration, and the number of gauche conformers in the structure, *N*_gauche_. The definition of the “radius of gyration” is context dependent and thus is not unique. For our purposes, which is to introduce a measure of compactness of C_14_H_30_, we adopt the simple definition
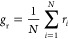
where *r*_*i*_ is the distance of atom *i* from the center of mass, and *N* is the number of atoms, which in the present case is 44. *g*_r_ is calculated as a function of time in MD calculations for a given total internal energy. The flexibility measure comes from the rate at which *g*_r_, the C–C distance distribution, or *N*_gauche_ change. The MD calculations start at the global minimum (GM) structure, a linear zigzag shape, shown in Figure 1. The rate of change by any of the three measures increases with the total energy.

The probability distribution for finding a C–C length of a particular value is now described in some detail. This measure is obtained by listing all the C–C lengths for each frame of a trajectory and making a histogram at a particular bin size. We then convert the histogram to a distribution by plotting the probability of being in a particular bin as a function of bin center. The resulting curve gives the probability of finding a C–C bond length per Å as a function of distance. When starting with the GM structure, all the C–C bonds are of similar length so that the probability distribution is a series of sharp peaks at the values of the C_1_–C_2_, C_1_–C_3_, ..., C_1_–C_14_ distances. With increasing time, these peaks broaden as the molecule becomes less ordered. The rate of this change also increases with the total energy of the molecule.

## Results

### Analyses of Flexibility

From Figure 1, we see that the ends of the molecule are methyl groups, CH_3_, and the interior CH_2_ groups have adjacent H_2_ groups trans to each other. All MD calculations are initiated from this minimum, and so this is the configuration just prior to the “first step”. We now summarize briefly what happens during the trajectories. At first, the energy provided to the trajectory is all placed as kinetic energy of the atoms. Within a few fs, the kinetic energy and potential energy exchange until each is approximately one-half of the total energy. During this time, the molecule barely changes from its original structure, so that the energy is still predominantly in the vibrational modes corresponding to the GM structure. In the first few 100 fs, the energy explores the anharmonic regions of the potential and the molecule starts to move away from its original structure. During the remainder of the 1.21 ps trajectory, more and more of the potential energy surface is sampled, leading to increased diversity in the structure and, because of diverse environments of the bonding, a broadening of the spectral features. These changes are dependent on the energy at which the trajectory calculated. For 5000 cm^–1^, there is hardly any change, whereas for 50,000 and 80,000 cm^–1^, the change approaches an “equilibrium” state; fluctuation still occurs, but the average structural measures change only slightly. We next turn to the measures of the structural diversity and then move to describe the accompanying spectral changes.

First, we present distributions of all C–C bond lengths obtained from MD trajectories at several total internal energies. These are NVE trajectories; however, as shown previously^12^ these correspond approximately to temperatures calculated using with the classical result that the average energy equals 126 RT for C_14_H_30_. Thus, the temperature in *K* is approximately 0.0114 times the total energy in cm^–1^; e.g., 50,000 cm^–1^ ≈571 K.

**Figure 2:**
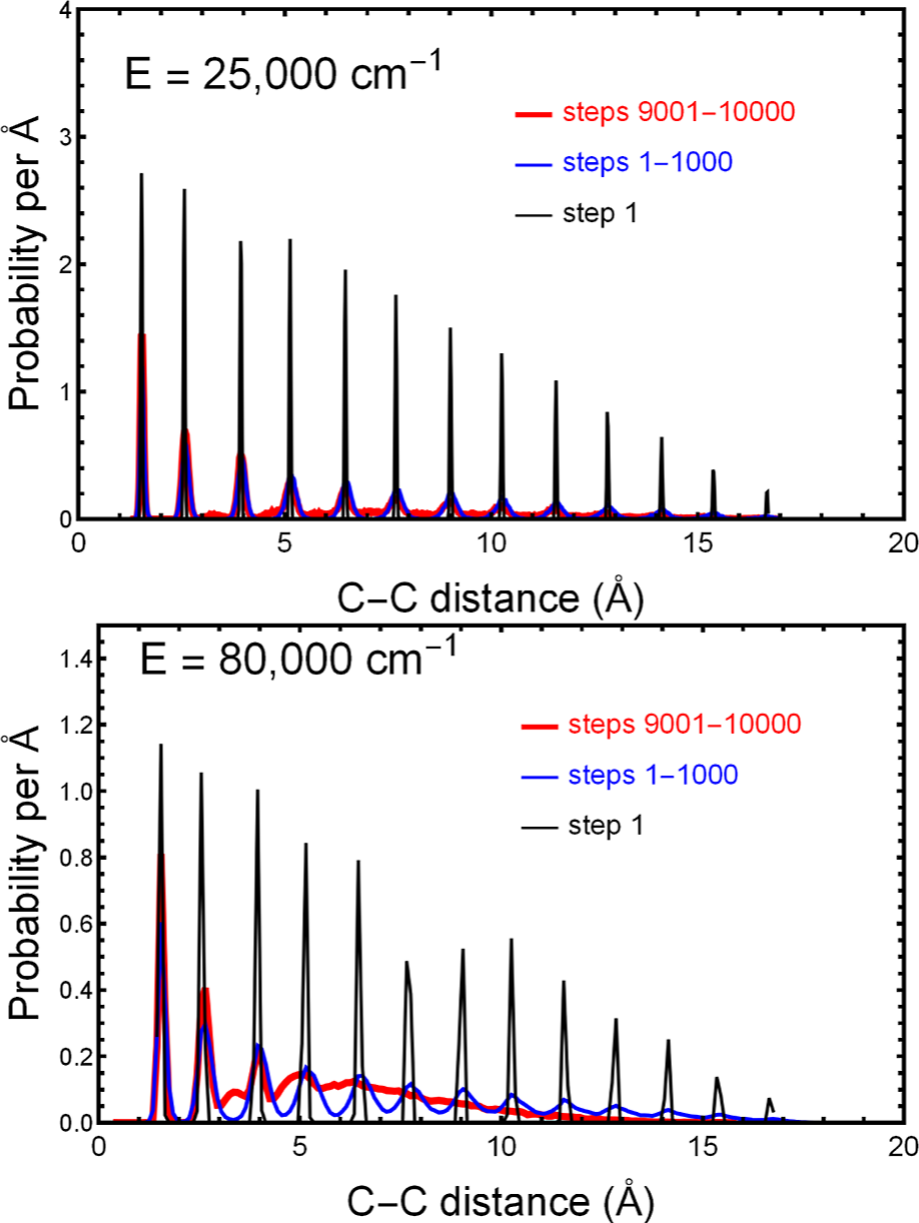
Probability per Å of finding a C–C distance in C_14_H_30_ is shown for two total energies and three time intervals, where 10,000 steps = 1.21 ps. For both energies, the distribution is sharp at the first step because nearly all the C–C distances are multiples of a common distance in the linear (global minimum) zigzag structure. With increasing time, these sharp features broaden, until, for 9001–10,000 steps, the distance distribution fills in the gaps between the original peaks. This broadening occurs to a greater degree and more rapidly at higher energies.

Figure 2 shows two C–C probability distributions, one for 25,000 cm^–1^ and one for 80,000 cm^–1^. Different colors correspond to different numbers of time steps in the trajectory. Every 10 time steps we record a “frame”, which corresponds to a printout of potential energy, coordinates, velocities, and gradients. The time between frames is 10 steps = 1.21 fs, and the total trajectory is 1000 frames or 10,000 steps or 1.21 ps. The black curves give the distribution at the end of ten steps, the blue curves are an average of steps 1–1000, and the red curves are the average of steps 9001–10,000. As can be seen, in both panels the probability distributions broaden with time, and they do this at a faster rate at the higher energy. (In the original 1000 frames, the near linear decline in the height of the spikes with C–C distance is a consequence of the linear decline in the number of nearest-neighbor, next-nearest-neighbor, etc., C–C distances.)

**Figure 3:**
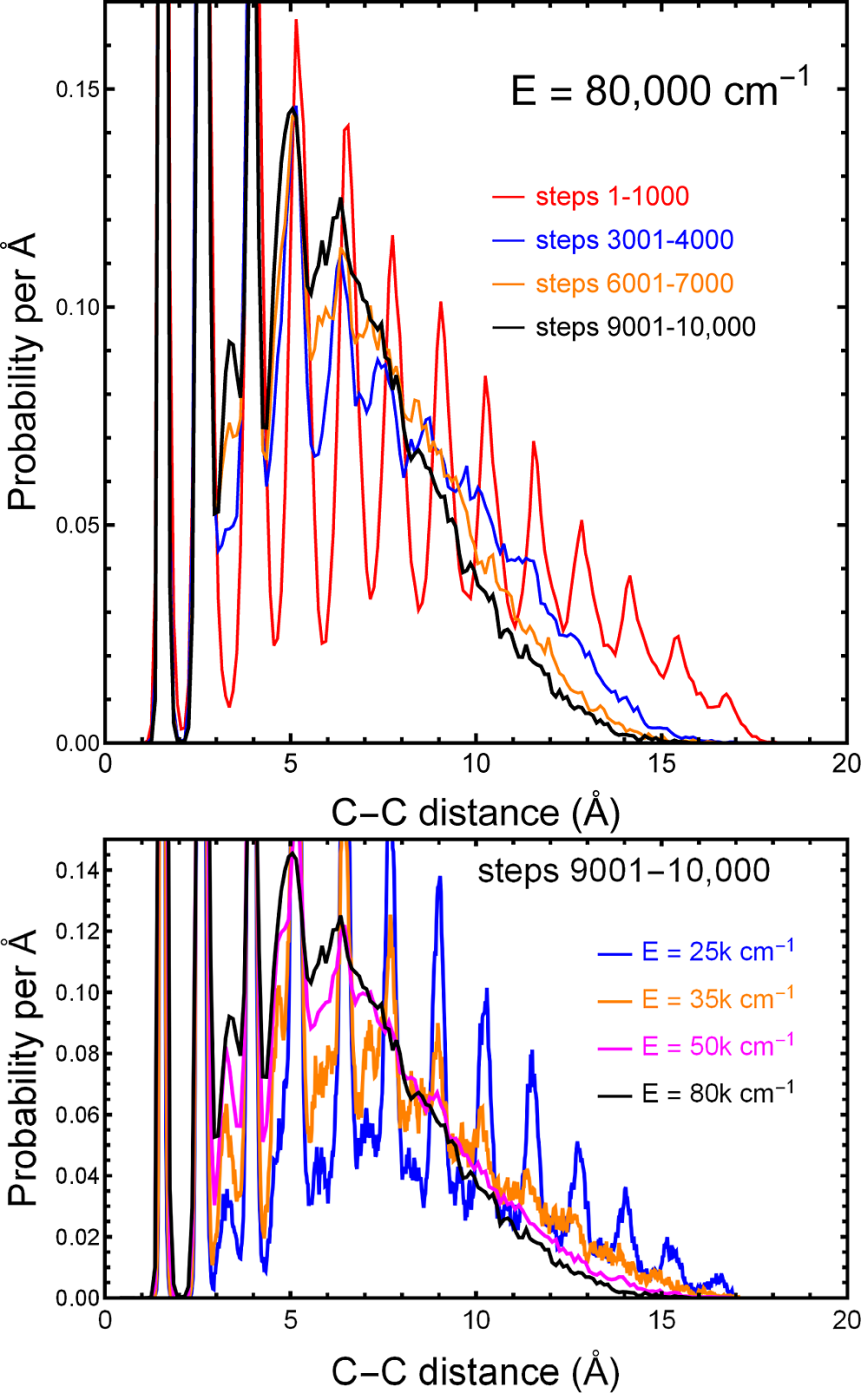
Expanded region of probability distributions such as those in Figure 2. Upper panel: the time evolution (10,000 steps = 1.21 ps) for *E* = 80,000 cm^–1^. Lower panel: the distributions averaged over the final 1000 steps for a range of total energies.

An expanded view is shown in Figure 3, where in the top panel we see curves for different times, and in the bottom panel we see curves for different energies all averaged over the last 1000 steps of the trajectory (the last 0.121 ps). In both cases, we used trajectories at 80,000 cm^–1^. In both cases the final distributions, depicted as black lines, show substantial intensity between the original peaks, confirming that the structure is much less uniform that it was at the start of the trajectory. From the lower panel, we see that the rate of the broadening of the peaks is a strong function of energy. In both cases, the correlation between C–C distances, while still present for the nearest 1–5 carbon atoms, is lost when the carbons are farther from each other.

**Figure 4:**
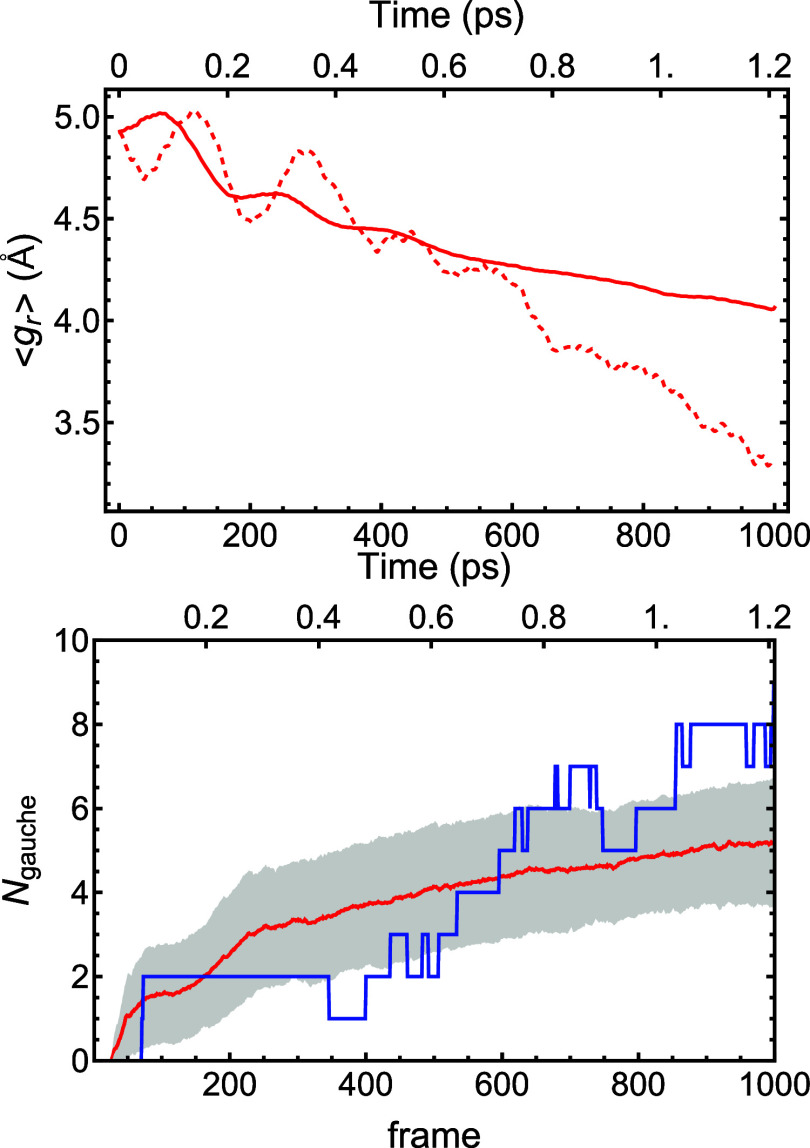
Upper panel: the radius of gyration, *g*_r_, as a function of frame (1000 frames = 1.21 ps) for trajectories with a total energy of 80,000 cm^–1^. Solid line: *g*_r_ is averaged over all 154 trajectories calculated for a total energy of 80,000 cm^–1^. Dashed line: a selected trajectory that tends toward a spherical shape at 1000 frames. Lower panel: the number of gauche bonds as a function of frame (1000 frames = 1.21 ps) for a trajectory with a total energy of 80,000 cm^–1^. Red: *N*_gauche_ averaged over all 154 trajectories calculated for a total energy of 80,000 cm^–1^. The shaded area shows ±1 standard deviation. Blue: *N*_gauche_ for the same selected trajectory as shown in the upper panel.

Another measure of the changing shape of the molecule with time and energy is the radius of gyration, *g*_r_. The top panel of Figure 4 shows both *g*_r_ for a single selected trajectory (dashed line) and for the average of *g*_r_ over 154 trajectories, all at 80,000 cm^–1^ (solid line). The selected trajectory shows a greater decrease in radius during the trajectory than the average; in fact, it was selected because it is one of several trajectories with final *g*_r_ values below 3.5 Å.

The variation of the number of gauche configurations with trajectory frame is shown in the bottom panel of Figure 4. The red line and gray areas (±1 standard deviation) give the average *N*_gauche_ over the same 154 trajectories at 80,000 cm^–1^, whereas the blue line gives *N*_gauche_ for the same selected trajectory in the upper panel. It should be noted that there are 11 possible dihedral angles in C_14_H_30_, so the maximum number of gauche configurations is also 11; if randomly distributed, the average would be 6, just a bit above the final average value.

**Figure 5:**
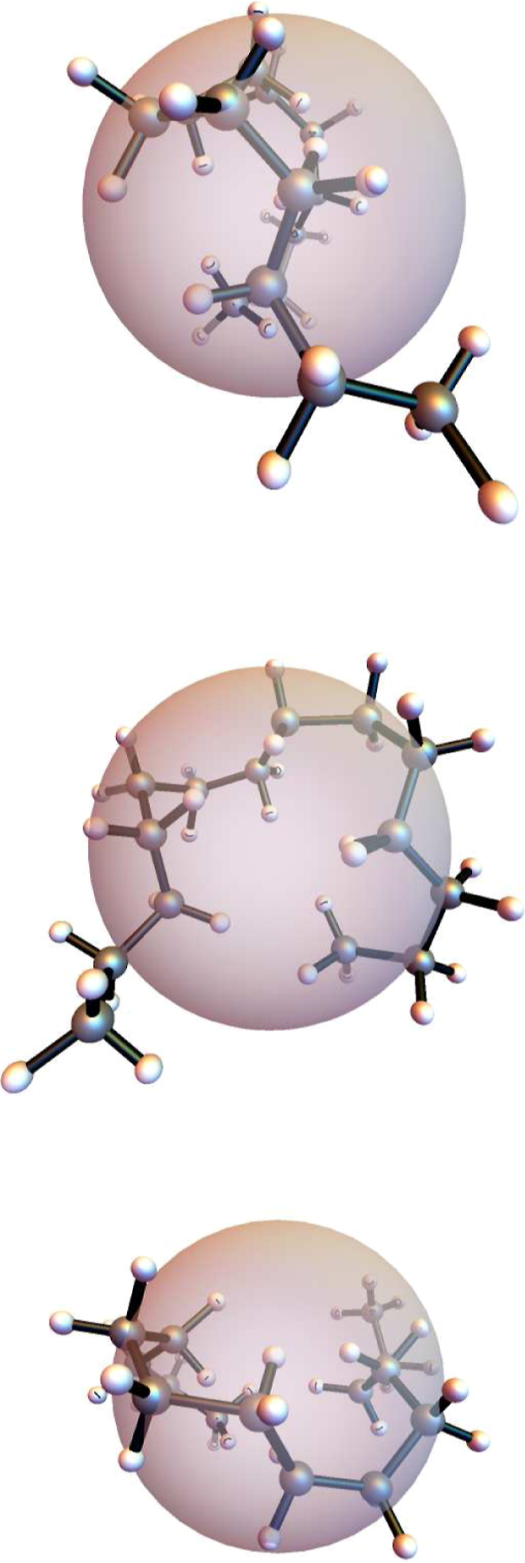
Views of the geometry of the selected trajectory at frame 1000 (time = 1.21 ps) when viewed, from top to bottom, along the *x*-, *y*- and *z*-axes, respectively. The sphere represents the radius of gyration.

The final C_14_H_30_ configuration of the selected trajectory in Figure 4 is shown from viewpoints along three orthogonal directions in Figure 5, where the sphere represents the radius of gyration. One can note that the molecule is considerable more compact than the starting geometry, shown previously in Figure 1.

### Power Spectra and Normal Mode Analyses

**Figure 6:**
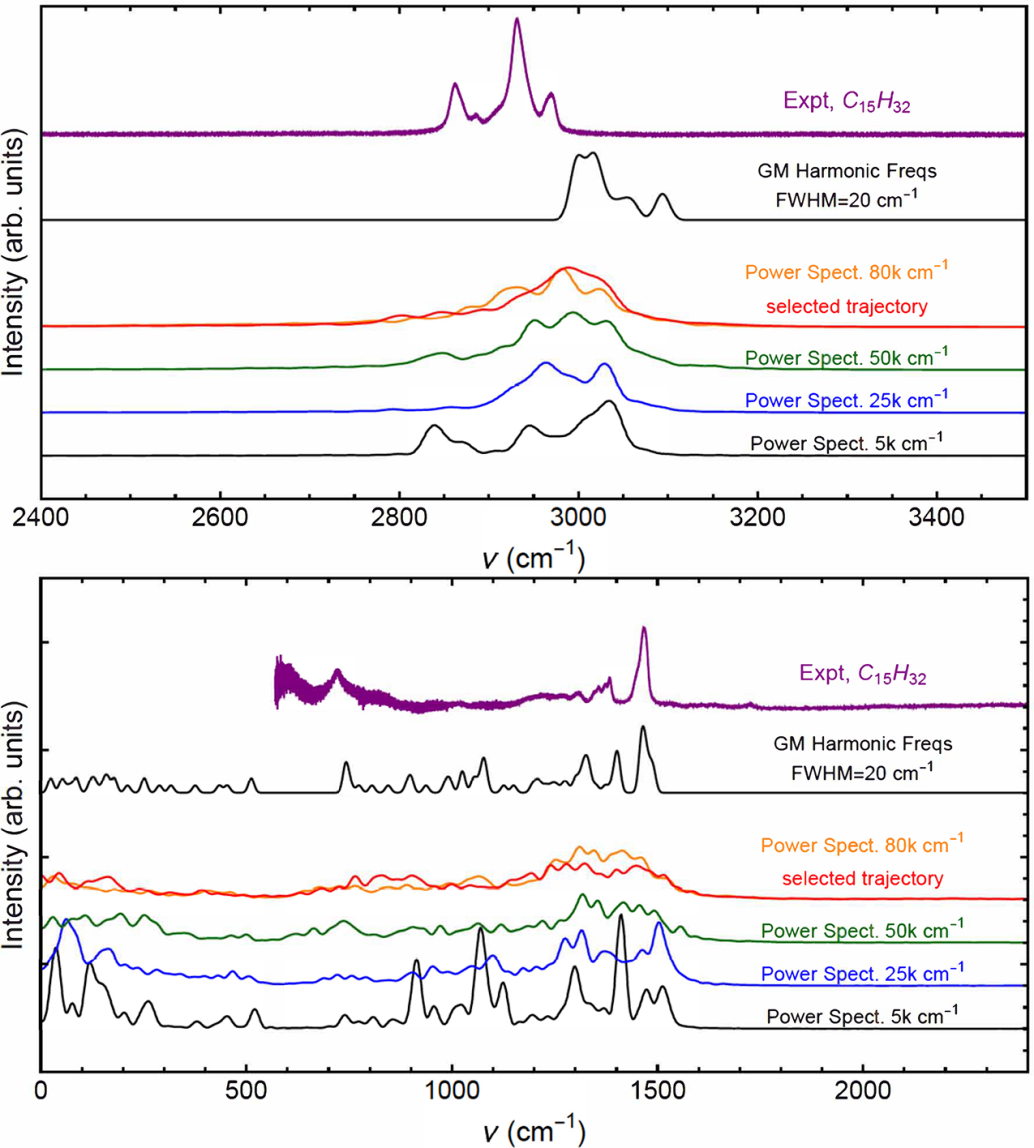
Upper panel: spectra of C_14_H_30_, including power spectra for indicated classical energies and PESs, the harmonic frequency spectrum, and an experimental spectrum of C_15_H_32_ in the spectral range of CH-stretches. Lower panel: corresponding spectra over a lower frequency range, including bends and CC stretches. The power spectrum of the selected trajectory, used in Figures 4 and 5, is shown in red.

Power spectra were calculated from a randomly chosen trajectory from those at each of four energies using SEMISOFT software,^16^ as described in the Methods section. The selected trajectory used in Figures 4 and 5 was also calculated. Figure 6 shows these spectra for a high frequency region that includes CH stretches (top panel) and for a low region that includes CC stretches and CC and CH bends (bottom panel). The spectra are shown at four different total energies. Also shown is a Gaussian-broadened (fwhm = 20 cm^–1^) stick spectrum of the normal modes calculated at the zigzag GM configuration. Finally, an experimental IR spectrum for a closely related molecule, C_15_H_32_, is provided for comparison.^4^ The spectrum and details of the measurements^20^ come from the Pacific Northwest National Laboratories database. The digital spectra of C_13_H_28_ and C_15_H_32_ are nearly identical, and, when plotted, they both look much like the spectrum of C_14_H_30_ found in ref (4) over the more limited spectral range in that paper. And since the experimental spectral range is much larger for C_15_H_32_ than C_14_H_30_, we chose the C_15_H_32_ spectrum for the comparisons with our calculations.

The resolution of a power spectrum depends not only on the inherent broadening of the vibrational bands but also on the duration of the trajectory—the longer the duration of the trajectory the finer the resolution provided by the technique. All trajectories used for the power spectra shown were the same duration (1.21 ps), so that changes in the spectra are attributable to a change in the inherent broadening and not to differences in how the power spectra were obtained.

Consider first the higher-frequency range corresponding the CH-stretches. We observe that the broadened harmonic band at the global minimum is up-shifted from experiment by roughly 50 cm^–1^ and does not have the same band shape. Of course the latter is perhaps strongly affected by the difference between the dipole-driven experimental IR spectrum and the power spectrum. The MD power spectra show significant broadening, even at 5000 cm^–1^ relative to the GM HO spectrum. This is presumably a consequence of the flexibility of the molecule, as discussed above. Also, the MD power spectra are downshifted from the HO one, as expected from anharmonicity in the MD PES. Further comment on this issue will be found below.

Next consider the spectral region from 0 to 2400 cm^–1^ in the lower panel. As seen, the GM broadened power spectrum lines up well with experiment in the bend band region of 1400–1500 cm^–1^. This is either fortuitous or a consequence of very little anharmonicity. Evidence for the latter is seen in the MD spectra which line up with HO one in position, unlike what was observed in the CH-stretch region. Again we do not make detailed comparisons with band shapes as these can be strongly affected by the differences between the power spectra and dipole-driven IR spectrum. In order to investigate the IR spectrum theoretically, we performed double-harmonic calculations of this spectrum, and we report these results next.

### B3LYP Double Harmonic Spectra

**Figure 7:**
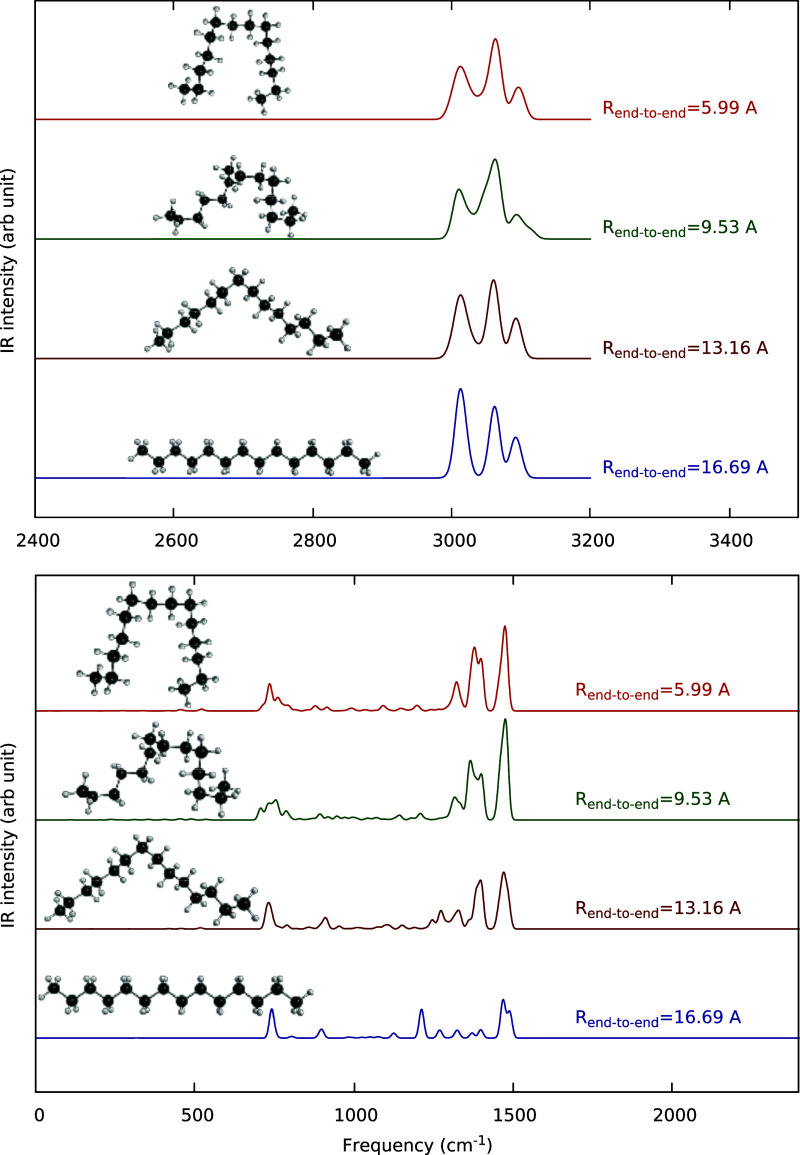
Double harmonic spectra of C_14_H_30_ at indicated structures in the high-frequency, CH-stretch, range 2400–3400 cm^–1^, upper panel, and in the frequency range from zero to the 2400 cm^–1^, which encompasses the CC stretches and bends, lower panel. Intensity values are given in Figure 8.

Direct double-harmonic spectra were calculated at roughly 200 stationary points at the B3LYP/pVDZ level. Selected results are shown in Figure 7 in two panels; the upper one for the CH-stretch region above 2400 cm^–1^ and the lower one over the large range from 0 to 2400 cm^–1^. As seen, the CH-stretch band shape is distinctly different for the global minimum compared to the other “nonlinear” structures. And, significantly, the band for those is close in shape to the experimental one, albeit upshifted by roughly 100 cm^–1^. A similar contrast is seen for the bend band in the range roughly 1200–1500 cm^–1^. And again, the band for the nonlinear structure is much closer in shape to experiment than the one for linear structure.

**Figure 8:**
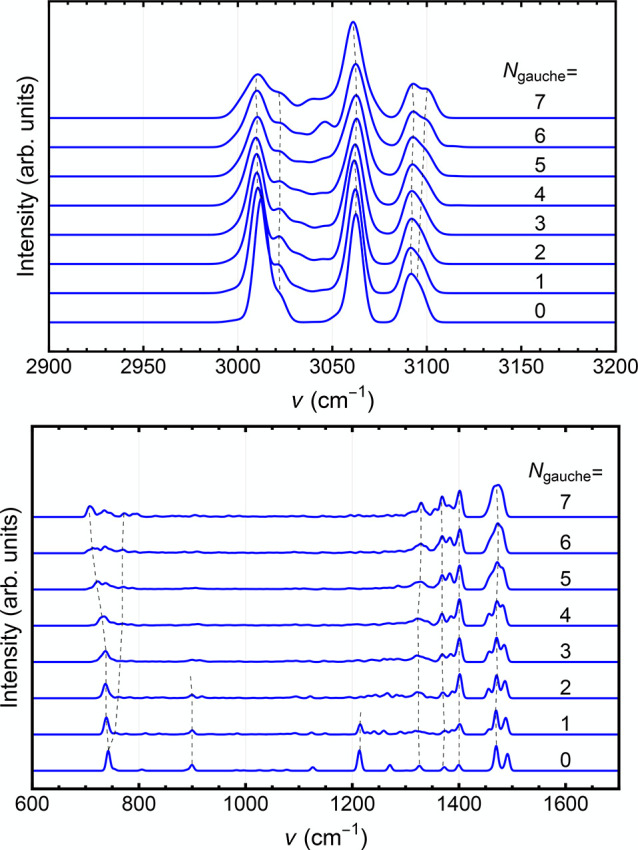
Double harmonic spectra of C_14_H_30_ as a function of the number of gauche-like structures. Upper panel: high frequency, CH-stretch region. Lower panel: low frequency, CC stretch and CC and CH bend region. The dashed black lines are a guide to the eye for features that are noted in the discussion.

In order to investigate these interesting findings, we plot the spectra again, but labeled by the number of Gauche configurations, *N*_gauche_, in Figure 8. The reason for using the number of gauche configurations is that these are what allow the molecule to twist and bend back on itself. Each *N*_gauche_ configuration introduces a ±120° twist in the ribbon-like shape of the molecule and brings the trans bonds into a gauched structure, causing the molecule to bend as well as twist. The upper panel of the figure shows the double harmonic spectra in the CH stretching region, while the lower panel gives the spectra in the CH and CC bending and CC stretching regions. Table 1 gives the potential energy, the C1–C14 distance, and the radius of gyration, averaged over 200 stationary points, as a function of *N*_gauche_. Although there is an energy penalty for each gauche configuration, it is very small compared to most total energies used in this study. Thus, at least for our reported energies above 10,000 cm^–1^, the transition from a trans to a gauche conformation is nearly random, and the final distributions will be governed mostly by entropy.

**Table 1:** Average Energy, C1–C14 Distance, and Radius of Gyration as a Function of the Number of Gauche-like Structures^a^

	*N*_gauche_	⟨*E*⟩ cm^–1^	⟨C1–C14⟩ (Å)	⟨*g*_r_⟩ (Å)	*N*_st.pt._
	0	0	16.7	4.93	1
	1	299	15.7	4.79	6
	2	590	14.2	4.58	13
	3	957	13.1	4.44	44
	4	1278	12.7	4.35	48
	5	1647	11.9	4.19	57
	6	2019	10.8	4.01	25
	7	2390	10.9	3.97	6
	8	2967	9.5	3.64	1

a The average is over the number of stationary points in the last column.

Next, we consider a simple quantum approach to investigate anharmonicty in the CH-stretch region, which is expected, based on upshift in the harmonic spectra compared to experiment.

### Local Mode Quantum Calculations of the CH-Stretch Energies

As noted above, anharmonicity is underestimated in the classical MD power spectra and not present, by definition, in the double-harmonic approximation. And there is evidence, i.e., comparison with experimental spectra, that this is significant for the CH-stretch band. (This is also well-known for XH-stretches.) To investigate this we use the local-mode model for XH-stretch modes applied to triatomic and tetraatomic molecules.^21,22^ In the present application, the CH_2_ group is the “triatomic” and, in the simplest version of this model, each CH-stretch is described by the one-dimensional (1D) Schroedinger equation
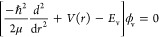
2where μ is the CH reduced mass, *r* is the CH bond length, *V*(*r*) is the 1D potential cut in the full potential, with all other atoms held fixed, and *E*_v_ and ϕ_v_ are the associated energy eigenvalue and eigenfunction, respectively. And this 1D Schroedinger equation applies to each CH stretch in CH_2_ or CH_3_. We solved this 1D Schroedinger equation for every CH stretch (30) for the four structures shown in Figure 7, using 1D discrete variable representation (DVR).^23^ The fundamental excitations of these local CH stretches are between 2873 and 2984 cm^–1^, in remarkable (and probably somewhat fortuitous) agreement with the experimental band between 2850 and 2975 cm^–1^.

**Figure 9:**
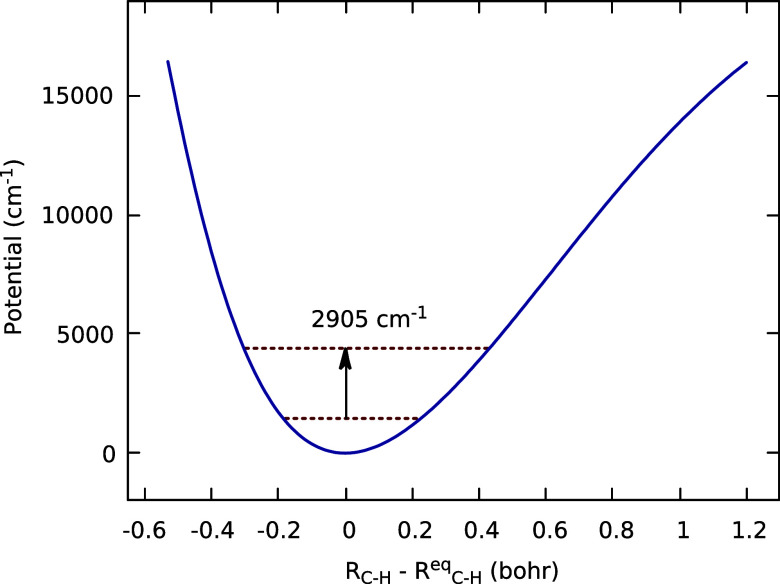
1D potential for a CH stretch from one of the CH_2_ groups in the global minimum structure and its fundamental excitation energy.

Figure 9 shows the 1D potential for the CH stretch from one of the CH_2_ groups in the global minimum structure, and clearly the 1D potential is “Morse-like”, lowering the fundamental excitation to 2905 cm^–1^ compared to the harmonic value of 3023 cm^–1^.

## Discussion

There are three broad conclusions from the analysis of shape and flexibility, based on consistent results from the analyses of the C–C probability distributions, the *g*_r_ data, and the *N*_gauche_ data. The first is that the molecule loses its stiff structure with time and becomes much more randomly distributed in space. The second is that the rate of this change is greater as the energy of the molecule increases. The third is that, although the structure becomes more random with time, the molecule, on average, retains a ribbon-like or tubular shape for the duration of most trajectories that we ran. For example, it does not on average form a helical structure of all gauche configurations, but rather approaches a state with an average of 6 of 11 possible gauche conformations. Of course, some individual trajectories, such as those shown in Figures 4 and 5, do fold more significantly, and some fold less.

We now turn to the important question of how these structural changes are manifest in the vibrational spectra of the molecule. The gas-phase experimental spectra have been considered by Williams et al.^4^ in infrared absorption at 298 K (shown in Figure 6) and by Lüttschwager and Suhm^7^ in the Raman spectrum using a jet nozzle expansion estimated to provide a vibrational temperature of 100–200 K.

Before discussing the comparisons with these experimental spectra, some remarks about the limitations of both the classical power spectra and the double-harmonic spectra are in order. First, note that harmonic zero-point energy (ZPE) of the global minimum is 90,914 cm^–1^. This means that at 80,000 cm^–1^, the highest total energy we consider, the energy in each CH-stretch mode is 635 cm^–1^; this is about one-third the ZPE for the CH-stretch of roughly 1800 cm^–1^. Thus, the MD simulations, even at 80,000 cm^–1^, do not sample the anharmonic region of these modes, nearly to the extent as would a quantum calculation. This is a well-known limitation of classical simulations. Second, the double-harmonic approximation, as the name implies, also does not describe anharmonicity of the potential. So in both instances the resulting spectra are not expected to be in quantitative agreement with experiment for the anharmonic CH-stretch modes, with possible compensating errors in the electronic structure method notwithstanding.

With these caveats in mind, consider the high-frequency power and experimental spectra (upper panel, Figure 6). As we see, there are perhaps four features in the experimental infrared spectrum; these correspond to CH stretching motions, of which there are 24 from the 12 CH_2_ groups and 6 from the 2 CH_3_ groups. Lüttschwager and Suhm attribute the highest frequency to the methyl asymmetric stretch, the next highest to the asymmetric methylene stretch, the next to the symmetric methyl stretch and the lowest to the symmetric methylene stretch. Williams et al. agree with the highest assignment but attribute the next highest to the symmetric methylene stretch. Our analysis concurs with that of Lüttschwager and Suhm. We note that the harmonic band is up-shifted relative to experiment by roughly 80 cm^–1^. Qualitatively this is the expected result owing to anharmonicity in the CH-stretches. In addition to being up-shifted from experiment, the band shape is somewhat different from the experimental one; however, both show a similar number of sub-bands. Of course the band shape difference could be due to the difference between the power and IR spectra. The classical power spectra calculated at 50,000 and 80,000 cm^–1^ are down-shifted from the harmonic one and also exhibit changed sub-band structure. The down-shifting is easily understood based on the increased classical anharmonicity at higher energies (and temperatures). These bands do move closer to experiment, but are still about 50 cm^–1^ up-shifted. A caveat about these differences is in order here. We are using DFT/B3LYP energies, and these are not expected to produce “spectroscopic” accuracy, i.e, to with a few cm^–1^ of the “exact” answer. So, overall we are pleased with the alignment of the high energy power spectra and the experimental IR one for the CH-stretch region on the spectrum.

The low-frequency spectrum (lower panel) between 1400 and 1600 cm^–1^ is attributed to CH-bending motions, particularly the scissors motion of the methylene groups at 1470 cm^–1^.^4^ The region from 800 to 1400 cm^–1^ is due to CC-stretching motions. Raman spectra have different selection rules than the IR power spectrum, but many modes are active in both spectroscopies. In the Raman spectrum,^7^ there are sharp peaks at approximately 1300, 1140, 1070, and 890 cm^–1^ that correlate well with peaks in the 5k cm^–1^ power spectrum and, to some extent, with the harmonic frequencies of the zigzag GM structure. The detailed experimental and theoretical paper of Lüttschwager and Suhm has the objective of identifying “hairpin” turns in the *n*-alkanes, and these authors provide convincing evidence that these occur in *n*-alkanes with 16 or more carbon atoms. Additional intensity for the hairpin turns is observed in the Raman regions from 175 to 275 cm^–1^, near 890 cm^–1^, and between 1075 and 1150 cm^–1^. For our carbon atom number of 14, we do not expect to see hairpin turns, but for the selected trajectory we do expect something similar. There are hints in the difference between the power spectrum of the selected trajectories, both at 80k cm^–1^, that there is more intensity in the first two of the expected regions. A somewhat surprising result is that the GM harmonic frequency distribution looks more like the experiment than the high-energy power spectra.

Perhaps the most obvious observation in comparing the power spectra at various energies is the correlation between the broadening of the spectral features with increasing energy and the previously described structural features that show broadening of the C–C probability distributions, decrease of the *g*_r_ and increase in *N*_gauche_. As noted earlier, while the resolution of the power spectra does depend on the conditions at which the trajectory was run, all of the trajectories used were run under the same conditions. Thus, the observed broadening of the power spectra with energy is due to changes in the molecule. Clearly, the details of the classical power spectra show a strong dependence on the internal energy, in contrast to the experimental IR spectrum for dodecane and smaller *n*-alkanes^3^ mentioned in the Introduction.

We note that another recent article by DelloStritto and Klein explores a different aspect of the flexibility of *n*-alkanes using molecular dynamics with a neural network potential.^2^ Specifically, they find that polyethylene chains with a 3_1_ overhand knot are weakest at the entrance and exit of the knot, where the C–C–C bond angles and lengths are strained. In order to be able to accommodate knots, the chains must be longer than about 50 carbon atoms, so these chains are considerably more malleable than ours of only 14 atoms.

We now turn to the double harmonic spectra of stationary point geometries shown in Figures 7 and 8. Figure 7 shows spectral changes for selected geometries with differing C1–C14 distances. While the top geometry resembles what might be called a “hairpin” turn, it is not the tight type of hairpin that is considered by Lüttschwager and Suhm.^7^ In higher frequency CH-stretch region, the spectrum starts to resemble that of experiment as the end-to-end distance decreases, while in the lower panel the starts to resemble that of the harmonic frequency distribution. We note that, for both panels, the difference between the blue spectrum at an end-to-end distance of 16.69 and that of the black harmonic GM frequency distribution in Figure 6, is due to inclusion of the transition dipole moments in the former; the frequencies are identical.

Figure 8 shows how the double harmonic spectrum changes with the number of gauche conformations. In the high-frequency region, the CH-stretch peaks change in intensity as *N*_gauche_ increases, with more prominence of the middle peak with increasing *N*_gauche_ as well as a filing in of the region between the lowest two peaks and a slight splitting of the highest frequency peak. In the low-frequency region, the peak near 750 cm^–1^, assigned to a collective rocking motion, broadens, shifts to lower frequency, and then splits up with increasing *N*_gauche_. The peak at 900, a collective CC stretching/rocking motion, cm^–1^ splits in two and then disappears. The peak at 1225 cm^–1^, due to CC stretching, disappears, the CC stretching peaks just at and below 1400 cm^–1^ bunch up and become more intense as the lower ones shift slightly to higher frequency, and the main peaks just below 1500 cm^–1^, due to the methylene scissors motions, split and broaden. While it is not always obvious why these changes occur, it is clear that the spectral features track the change in geometry.

The exploratory local mode approach we took for the CH-stretch appears promising. Further work using that and additional approaches will be done in the future. One approach, that allows for coupling of the stretch modes, uses the mass-scaled normal modes corresponding to the two CH-stretches, *Q*_s_ or *Q*_as_ and where the 1D cut is in the normal mode.^24^ Another approach is the local monomer one, where each CH_2_ and CH_3_ can be coupled together using a Hückel model.^25^

## Summary and Conclusions

We reported microcanonical molecular dynamics analyses of the flexibility of the *n*-alkane, C_14_H_30_, in the energy range from 5000 to 80,000 cm^–1^ (corresponding roughly to temperatures of 5–900 K). The dynamics made use of our recent machine-learned potentials.^12^ The analyses focus on the distributions of all C–C distances, the radius of gyration, and the number of gauche configurations as a function of the total energy. Classical power spectra were also reported over this energy range. In addition, density functional theory (B3LYP) double-harmonic IR spectra were computed at numerous minima and compared to experiment. From these, we conclude that the calculated IR spectrum at the global minimum is not relevant to the experimental one. Many changes in features of the double harmonic spectra smoothly track the number of gauche configurations. Finally, exploratory local mode quantum calculations of the CH-stretch fundamental were presented. These exhibit anharmonic down-shifts relative to the harmonic results and are in good agreement with the experimental CH-stretch range from 2850 and 2975 cm^–1^.

## Data Availability

The data used to create the plots in this study are available by contacting the authors.
